# Basophilic Response in Patients with Persistent Symptoms Attributed to Lyme Borreliosis Treated with Hydrolysed Arabinoxylan Rice Bran

**DOI:** 10.3390/medicina61122156

**Published:** 2025-12-03

**Authors:** Basant K. Puri, Gary S. Lee, Georgia Tuckey, Lisa Wyness, Reine Corley, Lucy Monaghan, Sari J. Arminen

**Affiliations:** 1 Cambridge Advanced Research, Cambridge CB21, UK; sariarminen@pm.me; 2 Department of Psychology, Neapolis University Pafos, 8042 Pafos, Cyprus; 3 Department of Psychology, University of Southampton, Southampton SO17, UK; g.s.lee@soton.ac.uk; 4 The Clinical Department, TBD Clinic, Worthing BN14, UK; georgia@tbdclinic.com (G.T.); lisa@tbdclinic.com (L.W.); reine@tbdclinic.com (R.C.); 5 Department of Physiotherapy, Isle of Wight NHS Trust, Newport PO30, UK; lucy.monaghan3@nhs.net

**Keywords:** arabinoxylan, basophil, borreliosis, fatigue, immunoceutical, tick

## Abstract

*Background and Objectives*: MGN-3/Biobran (BRM4, Lentin Plus or Ribraxx) is a natural, rice bran-derived arabinoxylan immunoceutical that modulates the adaptive immune response to viral infections. In response to bacterial infections, basophils act as “first responders” and are also associated with modulation of the adaptive immune response. The maturation of pluripotent CD34^+^ stem cells into basophils is supported by the cytokine interleukin-3 (IL-3). The aim was to test the hypothesis that modulation of the adaptive immune response in bacterial infection by MGN-3/Biobran entails a basophilic response. The tick-related disorder Lyme borreliosis was chosen as the disease model; tick bites are associated with cutaneous IL-3-mediated basophil recruitment. *Materials and Methods*: A three-month randomised double-blind placebo-controlled trial was conducted in patients with a history of borreliosis who were suffering from symptoms attributable to this disorder. The immunoceutical group received oral Biobran; the dosage for both groups was 1 g thrice daily. Both groups were matched for age, sex, and ethnicity. *Results:* A higher percentage of basophil count occurred in the immunoceutical group (*p* = 0.038). The final general linear model included the group (immunoceutical/placebo) and change in fatigue assessed by the 11-item Chalder Fatigue Questionnaire (CFQ) (*r*^2^ = 0.63; *p* = 0.0066). The change in basophil count was positively correlated with CFQ change (*r_s_* = 0.633; *p* = 0.020); only the immunoceutical group showed a positive correlation. *Conclusions*: These results support the hypothesis being tested. Basophils may modulate the adaptive immune response by acting as immunoregulatory cells. They can regulate the functioning of type 2 T-helper lymphocytes, enhance immunological memory, and present antigens to CD8 T lymphocytes. Further studies are needed to clarify potential mechanistic factors and the timing of this basophilic response.

## 1. Introduction

Lyme borreliosis is a tick-related zoonosis caused by members of the spirochaetal bacterial *Borrelia* species [1]. Clinical manifestations of Lyme borreliosis include dermatological disorders, neuroborreliosis, carditis, and arthritis [2]. Persistent symptoms attributed to Lyme borreliosis can include fatigue, pain, cognitive disturbance, and chronic arthritis [2,3]. Given the bacterial nature of its aetiology, it has been hypothesised that prolonged antibiotic treatment might help patients with such persistent symptoms [3,4]. However, long-term antibiotic treatment can be associated with several risks, including adverse side-effects, damage to the gastrointestinal microbiome, and, not least, potentially contributing to antimicrobial resistance [5]. We were, therefore, interested in the potential benefits of a natural immunoceutical, as an alternative to antibiotic treatment in patients with persistent symptoms attributed to Lyme borreliosis, and we initially considered MGN-3/Biobran. To understand the reasoning underlying this choice, it is helpful briefly to discuss the relevant aspects of the immune response to microbial infection, and then how the actions of this immunoceutical augment this immune response.

Host immunity is classically dichotomised into innate immunity, which is relatively fast acting and non-specific, and the phylogenetically younger adaptive immunity, which is relatively slower to act and more specific, and which can give rise to immunological memory [6,7]. Following microbial invasion, typically through a host epithelial/endothelial barrier, the innate immune response is activated. In contact with many of these barrier surfaces (such as in the skin, gastrointestinal tract, and lungs), as well as in the blood, are multi-branched mature dendritic cells. These dendritic cells have receptors that may detect pathogen-associated molecular patterns associated with the invading microbial pathogens. Such dendritic cells are able, then, to carry intact or degraded fragments of the pathogen to local lymph nodes. Here, the intact or degraded pathogens are presented to T lymphocytes of the adaptive immune system. Recognition by T lymphocytes causes T lymphocyte activation. In turn, this initiates the adaptive immune system. So, following infection, both innate immunity and adaptive immunity work together in humans.

Turning now to the immunoceutical, MGN-3/Biobran (known also as BRM4, Lentin Plus, and Ribraxx) is a natural, rice bran-derived arabinoxylan [8,9,10]. In a process involving enzymatic modification of partially hydrolysed defatted rice bran with fungal *Lentinus edodes* mycelial extract, the original hemicellulose is converted into an arabinoxylan form, in which a backbone of 1,4-linked β-d-xylopyranosyl moieties is left with arabinose side chains [8,9,11,12]. In terms of its cellular immunoceutical activity, it has been reported that MGN-3/Biobran induces dendritic cell maturation and enhances the capacity to activate T lymphocytes, with concomitant actions on the adaptive immune response [11,13,14,15].

There is in vitro evidence to support this use of MGN-3/Biobran. MGN-3/Biobran has been reported to inhibit the replication of human immunodeficiency virus-1 (HIV-1) in primary cultures of peripheral blood mononuclear cells (PBMCs) [8]. In vitro antiviral activity has also been demonstrated against severe acute respiratory syndrome coronavirus 2 (SARS-CoV-2) using the same immunoceutical [16]. In a recent age-matched controlled study on humans, PBMCs from eight subjects who had taken 1 to 2 g MGN-3/Biobran daily for one year showed an enhanced immune response against SARS-CoV-2 compared with the response of PBMCs taken from eight age-matched control subjects [17]. A six-week crossover clinical trial studying the potential benefit in the common cold reported that rice bran-derived arabinoxylan was associated with a non-significant reduction in the duration of common cold symptoms compared with rice bran [18]. Finally, in a clinical trial into influenza, administration of 500 mg MGN-3/Biobran daily for three months, compared with placebo, was associated with a reduced incidence rate and density of influenza-like illness [19].

However, all the above studies were in relation to viral infections. Given the bacterial nature of Lyme borreliosis and given that MGN-3/Biobran modulates the immune response, it was, therefore, decided to focus on those aspects of the immune response that are (i) particularly important in relation to bacterial infection and (ii) associated with modulation of the adaptive immune response. In respect of the second criterion, granule-containing white blood cells, known as granulocytes, are well established as “first responders”, and of these, two types are associated with modulation of the adaptive immune response [20]. These two types of granulocytes are eosinophils (containing granules that stain pink with haematoxylin and eosin) and basophils (containing granules that stain blue with haematoxylin and eosin) [20]. Eosinophilic responses are particularly associated with parasitoses and hypersensitivity reactions [21]. Basophilic responses, on the other hand, are indeed well established as being associated with bacterial infection [22,23,24]. The maturation of pluripotent CD34^+^ stem cells into basophils is particularly supported by the cytokine interleukin-3 (IL-3) [25]. Thus, the basophilic response also fulfils the first criterion. Therefore, it was hypothesised that modulation of the adaptive immune response in bacterial infection by MGN-3/Biobran entails a basophilic response.

Finally, the choice of those with persistent symptoms attributed to Lyme borreliosis as the patient group for testing this hypothesis needs further justification. As mentioned earlier, ticks can transmit bacteria, such as the *Borrelia* species, which are associated with Lyme borreliosis [26]. It is therefore noteworthy that non-human mammalian studies have demonstrated that tick bites are associated with cutaneous IL-3-mediated basophil recruitment [26,27,28]. Hence, it seemed reasonable to test the hypothesis that modulation of the adaptive immune response in bacterial infection by MGN-3/Biobran entails a basophilic response in patients with persistent symptoms attributed to the tick-related disorder Lyme borreliosis.

## 2. Materials and Methods

*Experimental design*. A three-month randomised double-blind placebo-controlled trial was conducted in patients with a history of borreliosis who were suffering from symptoms attributable to this disorder. The immunoceutical group received oral Biobran at a dosage of 1 g three times per day. The placebo group received an identically appearing isocaloric placebo preparation, which was also taken orally at a dosage of 1 g three times per day. The study took place in the UK, with the laboratory assays being carried out at Cambridge Advanced Research, Cambridge.

**Figure 1:**
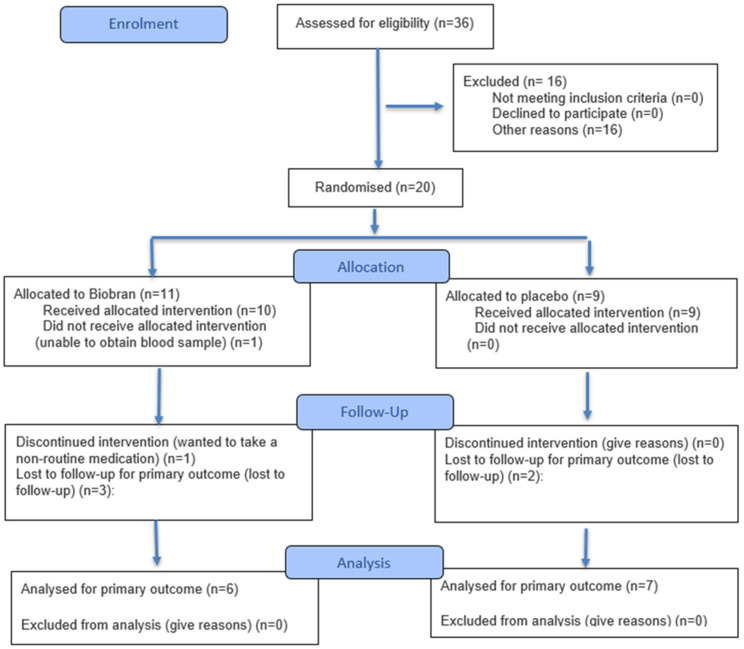
The CONSORT 2025 flow diagram for the study.

*Patients*. The inclusion criteria were the same as those of the Persistent Lyme Empiric Antibiotic Study Europe (PLEASE) [3], and were as follows. (1) Either male or non-pregnant, non-lactating female, age ≥ 18 years. (2) Complaints of musculoskeletal pain, arthritis, arthralgia, neuralgia, sensory disturbances (e.g., paraesthesiae or dysaesthesiae), or neuropsychological/cognitive disorders, which are either (A) temporally related to an episode of erythema migrans or otherwise proven symptomatic Lyme borreliosis (defined as within four months after erythema migrans as assessed by a physician, or positive biopsy, polymerase chain reaction (PCR) culture, or intrathecal *Borrelia burgdorferi* antibodies), or (B) accompanied by a positive *B*. *burgdorferi* immunoglobulin G (IgG) or IgM immunoblot, regardless of prior enzyme-linked immunosorbent assay (ELISA) IgG/IgM screening results. (3) Subjects must sign a written informed consent form. The exclusion criteria were a history of hypersensitivity to MGN-3/Biobran; having received > 5 days’ antimicrobial therapy during the previous four weeks; regularly taking MGN-3/Biobran during the previous four weeks; current enrolment in another clinical trial; currently receiving other antimicrobial therapy; and an inability to give full informed consent. The study was pre-registered with the ISRCTN registry, number 31318565 (ISRCTN originally stood for International Standard Randomised Controlled Trial Number). The CONSORT (Consolidated Standards of Reporting Trials) 2025 flow diagram [29] for the study is shown in Figure 1.

*Ethics*. Ethical approval for the study was received from the Research Ethics Committee of the Academy of Nutritional Medicine (approval number 20812-3). The study was carried out in accordance with the Declaration of Helsinki. All subjects gave informed written consent.

*Assessments*. Peripheral venous blood was collected directly into a 3-mL BD Vacutainer^TM^ plastic K2EDTA tube that contained ethylenediaminetetraacetic acid, acting as an anticoagulant, spray-coated on its interior surface (Becton, Dickinson and Company, Franklin Lakes, NJ, USA). The sample was then analysed using a Beckman Coulter DxH 520 Hematology System, which measured the white blood cell count using the impedance-based Coulter principle and then determined the basophil differential by combining this with direct optical (axial light loss) blue-wavelength measurement (Danaher Corporation, Brea, CA, USA). The assays were conducted blind to group status at baseline and follow-up.

At the baseline and follow-up appointments, each patient underwent clinical assessments using validated rating scales. Fatigue was assessed with the 11-item Chalder Fatigue Questionnaire (CFQ) [30] and the Fatigue Severity Scale [31]; autonomic function with the Refined and Abbreviated Composite Autonomic Symptom Score [32]; overactive bladder symptoms with the Overactive Bladder questionnaire [33]; and tinnitus with the Tinnitus Handicap Inventory [34]. These assessments were conducted blind to group status.

### Allocation Concealment and Blinding

The random allocation sequence was generated by a member of The Really Healthy Company, based in London, using a table of random numbers. A simple randomisation method, that is, without stratification or blocking, was employed, which generated two categories, namely “A” and “B”. Two sets of sealed sachets were supplied by Daiwa Pharmaceuticals, Japan, namely active and placebo. Prior to and during the whole duration of the study, the identity of these two types of sachets was unknown to anyone outside of Daiwa Pharmaceuticals. In particular, the person generating the “A” and “B” categories was blind to the identities of “A” and “B”. Similarly, all the participants and all the researchers were also blind to the identities of “A” and “B” prior to and during the study. The personnel who enrolled and those who assigned participants to the interventions did not have access to the random allocation sequence.

*Statistical analyses*. Two-tailed independent samples testing was conducted using the distribution-free Mann–Whitney *U* test. Fisher’s exact probability test was used to analyse a two-by-two contingency table. Multiple regression was conducted using the step-up procedure for entry of putative predictor or explanatory variables. Post hoc correlations were calculated using the distribution-free Spearman product-moment rank correlation coefficients. These statistical tests, as well as graphical plotting, were carried out using the programmes R v. 4.2.1 (The R Foundation for Statistical Computing, Vienna, Austria), JASP 0.19.3 (The JASP Team, Amsterdam, The Netherlands), stats v. 4.4.1, flexplot, and ggplot2 v. 3.5.2 [35,36,37,38].

## 3. Results

A total of 13 subjects took part in the whole study. Six were in the immunoceutical group and seven in the placebo group. The median age of the immunoceutical group (48.5 years) did not differ significantly from that of the placebo group (49.0 years, interquartile ranges (IQRs) 16.8 years and 12.5 years, respectively; *U* = 13, *p* = 0.283). Similarly, the two groups were matched for both sex (immunoceutical: one male; placebo: all female; *p* = 0.462) and ethnicity (all the subjects were White Caucasian).

**Figure 2:**
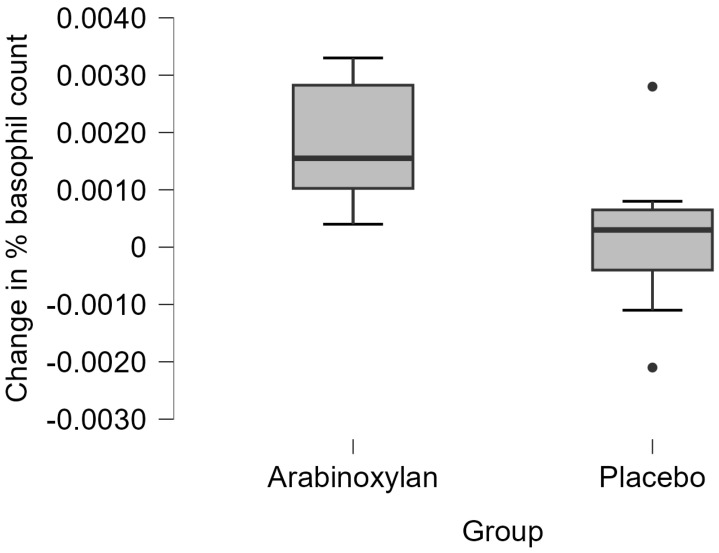
Percentage changes (three-month follow-up minus baseline) in basophil counts in the two groups. The thick horizontal line in each box represents the median, while the upper and lower horizontal boundaries of each box represent the upper and lower quartiles, respectively.

The median change in the percentage basophil count in the immunoceutical group (0.00155, IQR 0.00180) was higher than that in the placebo group (0.00030, IQR 0.00105; *U* = 36, *p* = 0.038). These findings are shown as boxplots in Figure 2.

The final general linear model included the CFQ change and the group (*r*^2^ = 0.63; *df* = 2, 10; *p* = 0.0066). The significant coefficients (omitting the intercept data) are shown in Table 1

**Figure 3:**
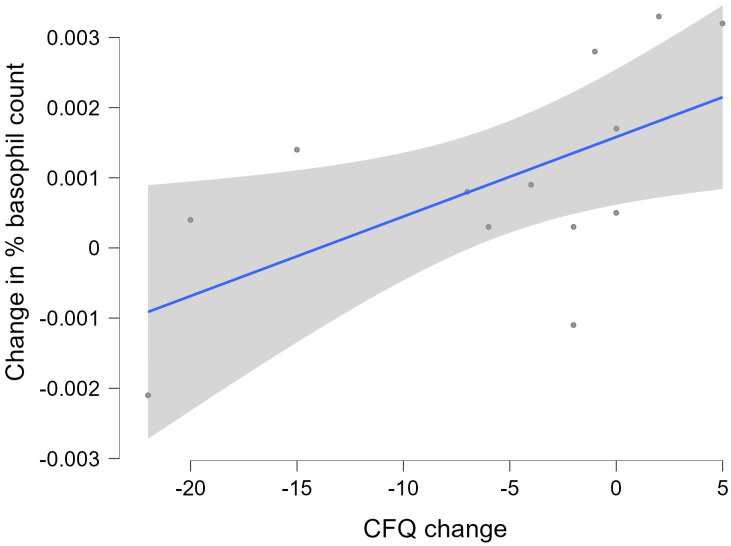
Change (three-month follow-up minus baseline) in the basophil count versus change (three-month follow-up minus baseline) in scores on the Chalder Fatigue Questionnaire (CFQ). The shaded area indicates the 95% confidence interval of the linear regression line.

Based on the general linear model findings, a post hoc analysis showed that the change in basophil count was positively correlated with the change in CFQ (*r_s_* = 0.633; *p* = 0.020). The corresponding scatter plot, linear regression line, and its 95% confidence interval are shown in Figure 3.

Dichotomisation of these correlational data by group showed that, while there was no significant relationship between the two variables for the placebo group, the immunoceutical group showed a positive correlation (*r_s_* = 0.886; *p* = 0.033).

## 4. Discussion

The trial showed that participants who received the hydrolysed arabinoxylan rice-bran supplement experienced a statistically significant increase in circulating basophils compared with placebo (median change = 0.00155 vs. 0.00030; *p* = 0.038). Although the *p*-value did not reach the conventional “highly significant” threshold of 0.01, it still indicated that the observed difference is unlikely to result from chance alone (roughly a 1-in-26 probability of a false-positive result). In practical terms, the immunoceutical group exhibited roughly a five-fold larger rise in basophil percentage, a magnitude that aligns with the biologically plausible mechanism we proposed—namely, that the supplement augments the IL-3-driven basophil recruitment that follows tick exposure. Importantly, this modest but reliable shift was accompanied by a parallel improvement in fatigue scores (CFQ change), suggesting that even a relatively small augmentation of basophils may translate into clinically meaningful symptom relief for patients with persistent Lyme-related fatigue. Future work with larger cohorts will be needed to confirm the effect size and to determine whether the basophil increase is a driver of symptom improvement or a correlative marker of broader immune modulation. It is noteworthy that basophils may modulate the adaptive immune response by acting as immunoregulatory cells [39,40]. Importantly, they can regulate the functioning of type 2 T-helper lymphocytes [41,42]; enhance immunological memory [23]; and present antigens to CD8 T lymphocytes [43].

As mentioned above, the basophilic response in the patient group was associated with improvement in symptoms of fatigue, as measured by the CFQ. To the best of our knowledge, there is no previous description of an association between a basophilic response and lower levels of fatigue. In addition to the possibility of such a direct association, there may also exist an indirect association, as follows. Post-exertional fatigue/malaise is a relatively common symptom in the patient group studied [44]. In a rat model of prolonged exercise, cerebral glutamate was found to be reduced after engaging in treadmill running until exhaustion [45] (Glutamate was reduced throughout the brain in previously trained rats, while a reduction in this neurotransmitter was confined to the striatum in rats that had not been trained.) Recently, it has been reported that MGN-3/Biobran modulates glutamatergic burst activity in human-induced pluripotent stem cell-derived neurones and astrocytes [12]. Given that MGN-3/Biobran also causes a basophilic response (the present study), the possibility of the above indirect association follows. It should also be noted that there is a small number of studies relating to arabinoxylan and fatigue. In their three-month study of patients with chronic hepatitis C virus infection, Salama et al. reported that, while 48% of 21 patients randomised to treatment with pegylated interferon plus ribavirin experienced fatigue, this was the case in none of 16 patients treated with pegylated interferon plus MGN-3/Biobran [46]. Kim et al. conducted a non-randomised eight-week trial in patients newly diagnosed with malignant tumours [47]. The active group comprised 10 patients who received a cereal-based oral nutritional supplement that included arabinoxylan-rich fermented rice bran, as well as black rice. By the end of the fourth week, the active group was significantly less fatigued than at baseline, while at the end of the eighth week, they were significantly less fatigued than the control group [47]. In their narrative review of the clinical applications of rice bran arabinoxylan, Ooi et al. reported that the effect of arabinoxylan on symptoms of fatigue in patients with chronic fatigue syndrome is unclear [10].

It is important to consider the distinction between the basophilic response to tick attachment and documentation of borrelial or another pathogen invasion. Arguably, a basophilic response to tick attachment per se, even in the absence of transmission of pathogens, might occur, given the multitude of chemically and biologically active substances secreted by ticks during the attachment and feeding processes. On the other hand, the effect of this distinction would be mitigated by the randomised, double-blind, placebo-controlled design of this study. Nevertheless, it would be good if detailed studies were to be carried out into the basophilic response to borrelial infections per se in both humans and non-human mammals.

### Limitations

A limitation of this study is that it did not directly address the mechanism(s) by which MGN-3/Biobran may give rise to a basophilic response. Moreover, the timing of this basophilic response needs clarification; there was just one follow-up assessment, at three months. Furthermore, treatment with MGN-3/Biobran and improvement in CFQ scores accounted for 63% of the variance of the change in basophil levels; thus, 37% of the variance was unaccounted for, and further possible variables need to be considered, including, as mentioned, potential mechanistic factors. Future studies should be conducted to address these issues.

## 5. Conclusions

The nutritional product MGN-3/Biobran has immunoceutical actions in adults. The present study suggests that modulation of the adaptive immune response by this rice bran-derived arabinoxylan involves a basophilic response. Further studies are needed to clarify potential mechanistic factors and the timing of the basophilic response to this immunoceutical and the role of basophils in tick- and vector-borne infections, such as Lyme disease, in animal studies and human subjects. Further studies using different doses are also required; a dose–response relationship will help determine the optimum dose for the immunomodulatory effect of this immunoceutical.

## Figures and Tables

**Table 1:** General linear model coefficients.

	Estimate	Standard Error	*t*	*p*
Change in CFQ	0.000111	0.000036	3.103	0.011
Group (placebo)	−0.001560	0.000580	−2.688	0.023

## Data Availability

The raw datasets generated and analysed during the present study are not publicly available in order to protect patient confidentiality. Case report forms in paper form are safely and securely stored. The data were transferred and anonymised to R and R-based programmes for statistical analyses. The data files are stored by C.A.R. on a server dedicated to research. The data are available upon reasonable request to the corresponding author.

## Abbreviations

The following abbreviations are used in this manuscript:

CFQ	Chalder Fatigue Questionnaire
CONSORT	Consolidated Standards of Reporting Trials
ELISA	Enzyme-linked immunosorbent assay
HIV	Human immunodeficiency virus
Ig	Immunoglobulin
IL-3	Interleukin-3
IQR	Interquartile range
ISRCTN	International Standard Randomised Controlled Trial Number
PBMC	Peripheral blood mononuclear cell
PCR	Polymerase chain reaction
PLEASE	Persistent Lyme Empiric Antibiotic Study Europe
SARS-CoV-2	Severe acute respiratory syndrome coronavirus 2
